# Supplementary material for: The comparison of cancer gene mutation frequencies in Chinese and U.S. patient populations
Source: Nat Commun. 2022 Sep 26;13:5651. doi: 10.1038/s41467-022-33351-4 (PMC9512793; doi:10.1038/s41467-022-33351-4)
Supplement: Supplementary file 1 — Supplementary Information [file 41467_2022_33351_MOESM1_ESM.pdf]

The comparison of cancer gene mutation frequencies in Chinese and U.S. patient populations

Fayang Ma, Kyle Laster, Zigang Dong*

- **Supplementary Fig. 1 ~ Fig. 5,**
- **Supplementary Table 1**

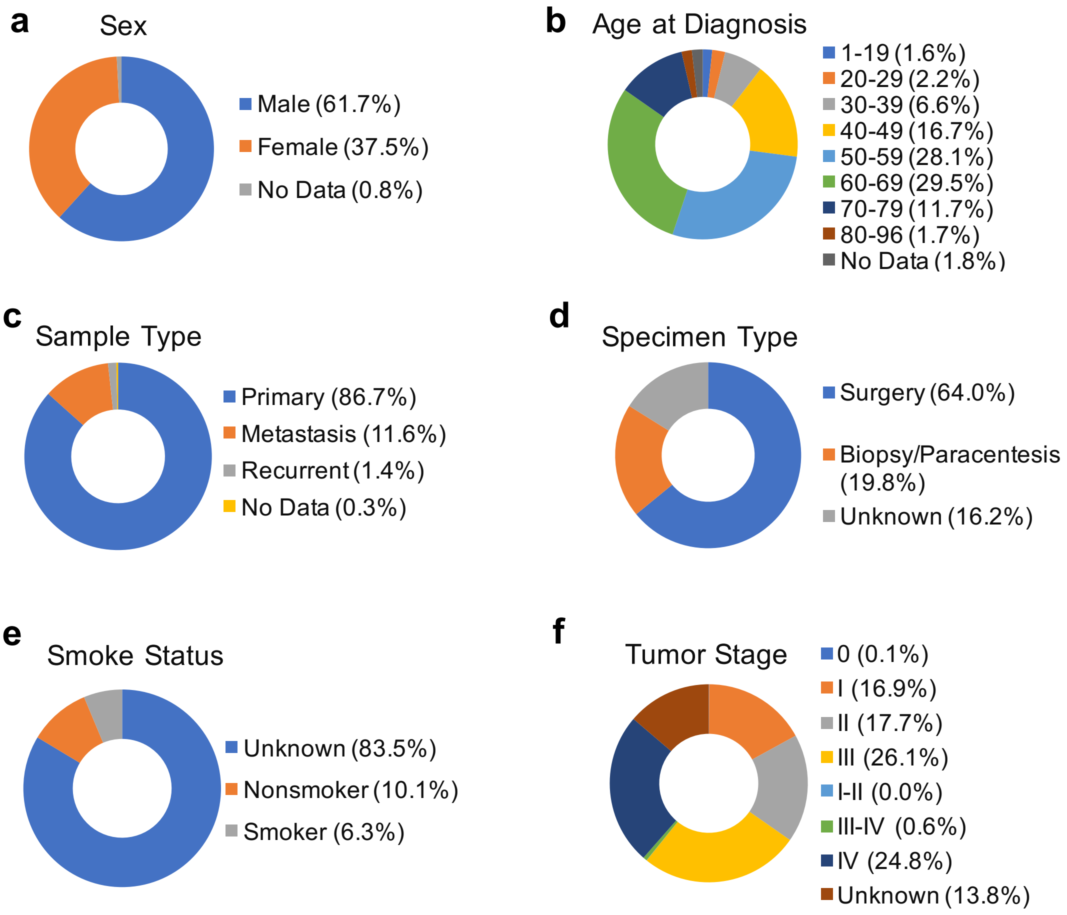

Supplementary Fig. 1 The demographical and clinical data of included Chinese cancer patients. a ~ f The demographic data and clinical information were summarized for the 11,948 cancer patients, the sequencing profiles of which were utilized to calculate the epidemiological weighted mutation rates of cancer genes in the China pan-cancer analysis. Source data are provided as a Source Data File.

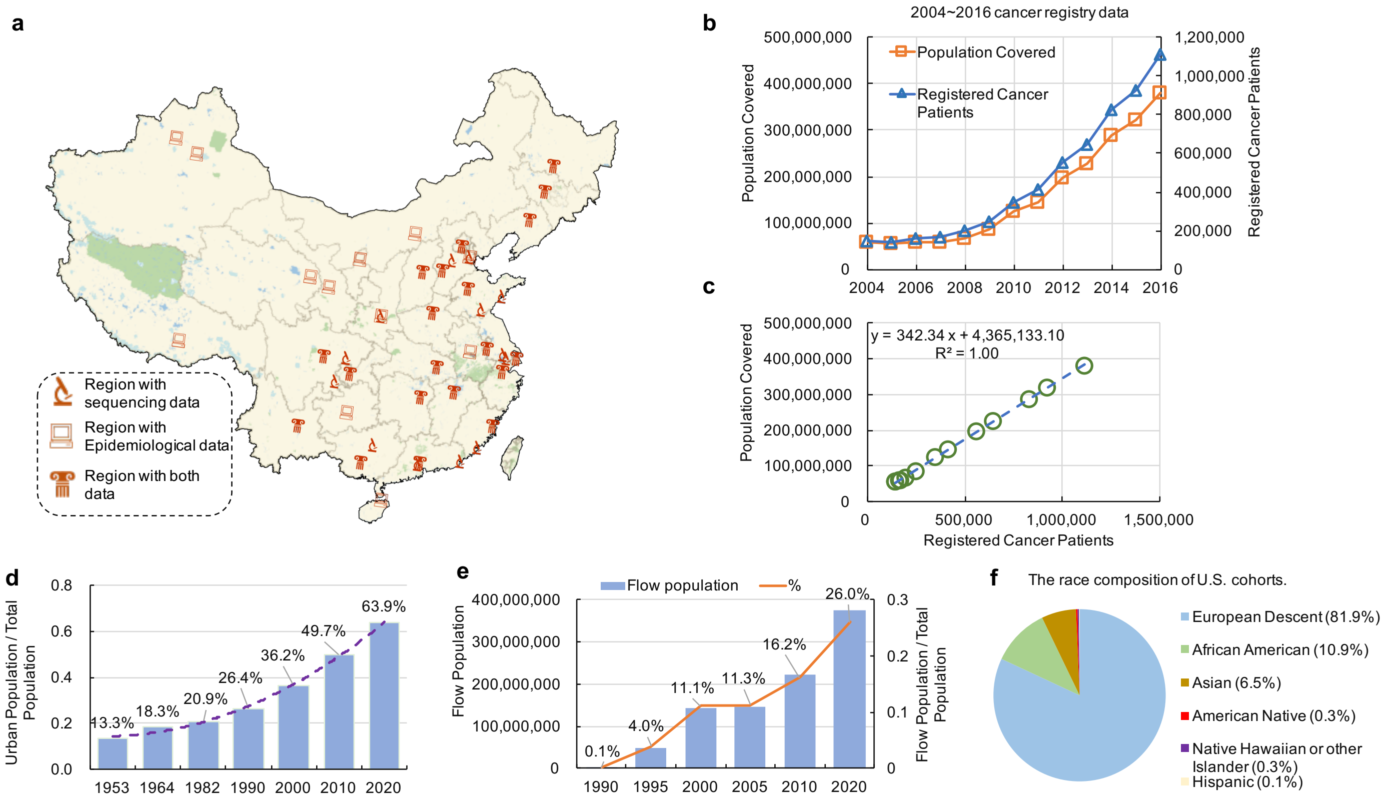

Supplementary Fig. 2 Supporting information for the sequencing and epidemiological data in CN cohorts. a A map of China illustrating the nation-wide distribution of cancer registries from which the epidemiological data used for mutation rates weighting were obtained (represented with the Computer icon), the tumor samples from which the mutational profile were sequenced (represented with the Microscope icon), and the places with both cancer registry and tumor sample (represented as the Pillar icon). **b** The number of registered cancer patients and corresponding population covered from 2004 to 2016 were summarized from the series of "China Cancer Registry Annual Report". **c** The number of registered cancer patients were highly and positively correlated with the population covered in the 13-years range (n = 13, Pearson *r* = 0.9997, 95% confidence interval 0.9990 ~ 0.9999, *p* < 0.0001). **d** The proportional estimates of urban population in total population derived from the seven population censuses carried out in 1953, 1964, 1982, 1990, 2000, 2010, and 2020, respectively (Data from China National Bureau of Statistics). **e** The number of flow population and corresponding percentage in total population in 1990, 1995, 2000, 2005, 2010, and 2020, respectively (Data from China National Bureau of Statistics). **f** From the 139 studies included in the U.S. cohorts, 11,526 cancer patients with known ancestry background were presented in the pie chart. Source data are provided as a Source Data File.

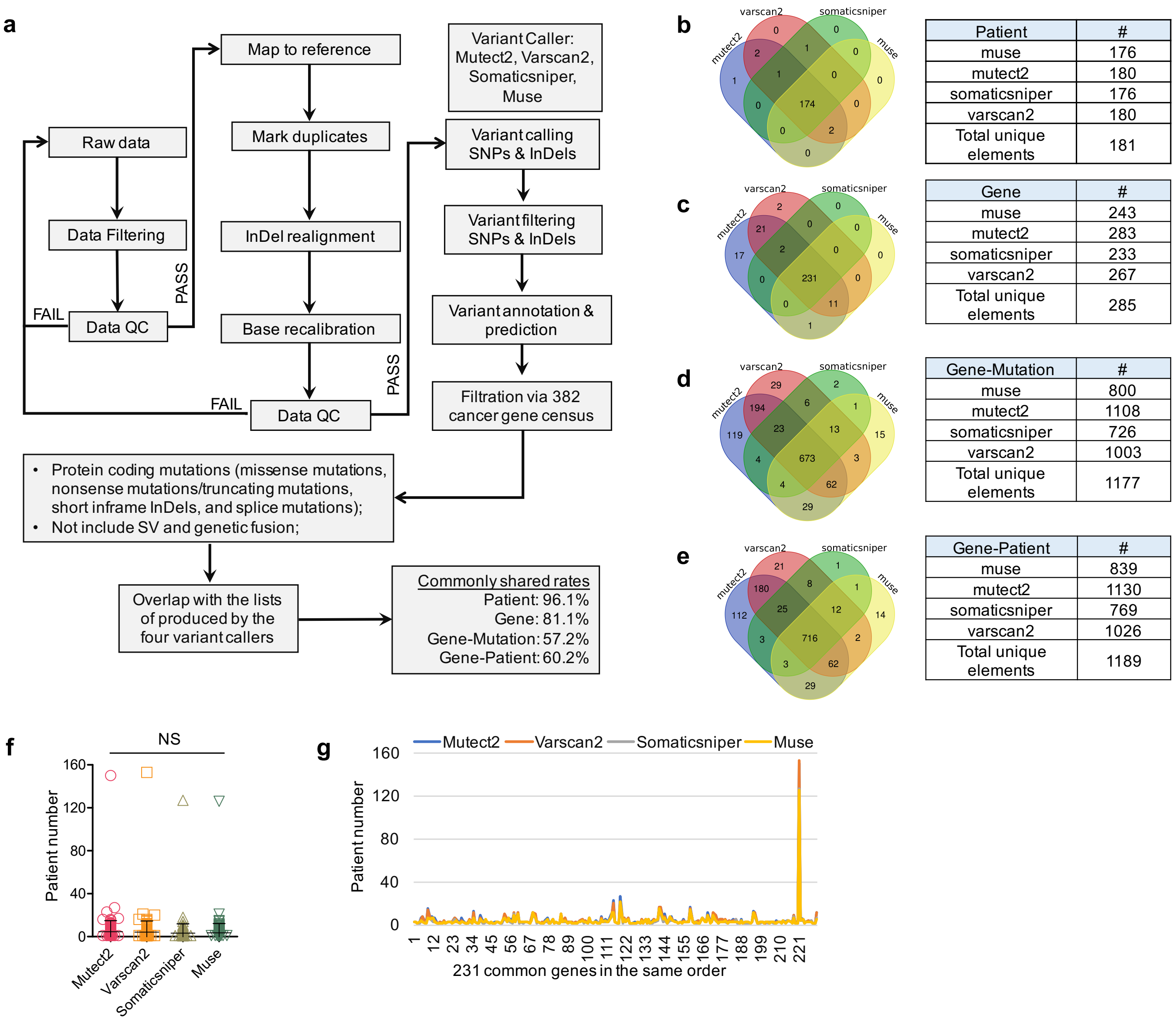

Supplementary Fig. 3 The performance evaluation of the four variant callers. a The variant calling workflow in which the same raw sequencing data (n = 181 patients) was processed via four different variant callers (Mutect2, Varscan2, Somaticsniper, and Muse). **b ~ e** The result lists obtained from (a) with respect to mutated patient, mutated gene, gene-mutation, mutated gene-patient were overlapped to produce the corresponding commonly shared rates among the four variant callers. **f** The patient number corresponding to each of the 231 commonly shared mutated genes (c) produced from four variant callers were compared and showed no statistically significant differences, n = 231 for all the four groups, Mean ± SE, p = 0.4531 (one-way ANNOVA, two-tailed, F = 0.8759; Bartlett's test for equal variances, Bartlett's statistic [corrected] = 16.54, p = 0.0009; the results of Dunnett's multiple comparison test showed the differences among the four datasets were not significant). **g** The patient number corresponding to each of the 231 genes were generally in the same trend among the four variant callers. Source data are provided as a Source Data File.

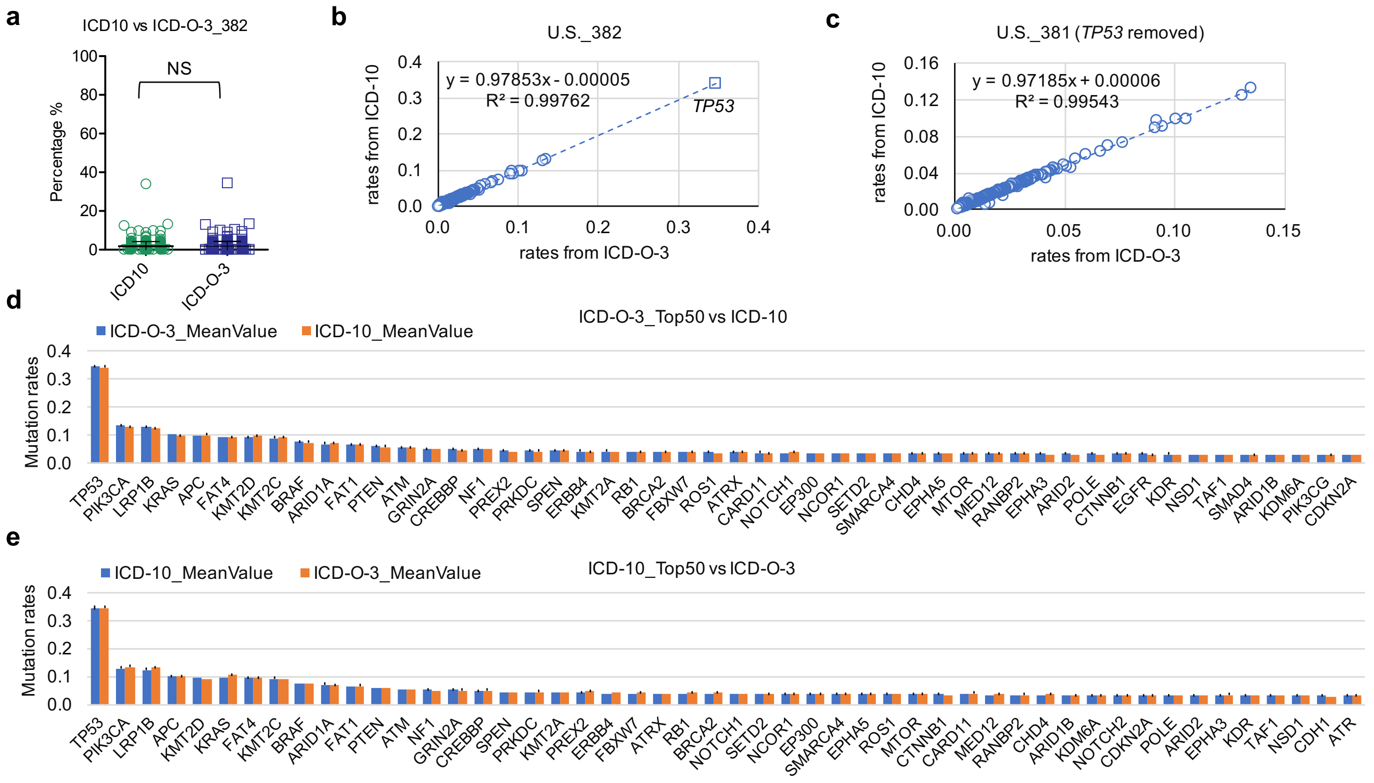

Supplementary Fig. 4 The consistency between the two data sets produced from ICD10 and ICD-O-3. **a** No statistically significant differences were observed between the ICD-10 (n = 18,584 patients) and ICD-O-3 (n = 19,181 patients) produced mutation rates with respect to the 382 cancer genes, *p* = 0.7964 (unpaired *t* test, two-tailed, *t* = 0.2581, *df* = 762; *F* test: *F* = 1.042, *DFn* = 381, *Dfd* = 381, *p* = 0.6897), ICD-O-3_ Mean ± SE = 0.01858 ± 0.001233 (N = 382 genes), ICD10_ Mean ± SE = 0.01813 ± 0.001208 (N = 382 genes), 95% confidence interval -0.003828 ~ 0.002937. **b** The mutation rates of the 382 cancer genes were highly and positively correlated (n = 382 genes, Pearson *r* = 0.9988, 95% confidence interval 0.9985 ~ 0.9990, *p* < 0.0001, two-tailed) . **c** After *TP53* was removed the correlation between ICD-10 and ICD-O-3 produced mutation rates were still highly correlated (n = 381 genes, Pearson *r* = 0.9977, 95% confidence interval 0.9972 ~ 0.9981, *p* < 0.0001, two-tailed). **d ~ e** The top50 of the 382 cancer genes produced from the two classification system ICD-10 (n = 18,584 patients) and ICD-O-3 (n = 19,181 patients) were compared and showed rather equivalent mutation rates. Mean ± Error, error bars represent the 95% confidence limits determined through simulated samples (n= 2, 000 independent Poisson distributed computational samples with the calculated mutation proportion as the central value), and measure of centre (bar levels) represent mean of simulated mutation proportions. Source data are provided as a Source Data File.

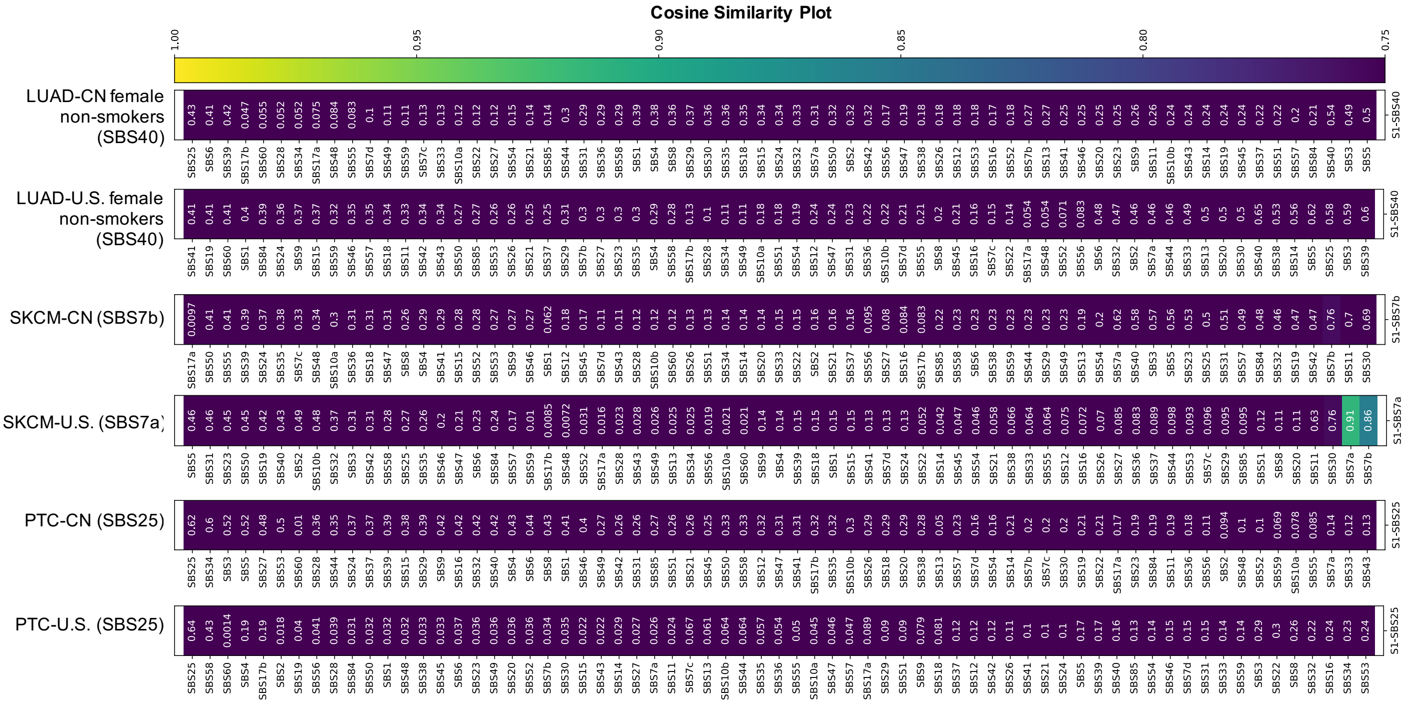

Supplementary Fig. 5 The Cosine similarity plots of lung adenocarcinoma, skin cutaneous melanoma, and papillary thyroid carcinoma in CN and U.S. cohorts. The Cosine Similarity Plot identified the mutation signature for LUAD-CN female non-smoker (SBS40), LUAD-U.S. female non-smoker (SBS40), SKCM-CN (SBS7b), SKCM-U.S.(SBS7a), PTC-CN (SBS25), and PTC-U.S. (SBS25). The corresponding Cosine Similarity values were 0.54, 0.65, 0.76, 0.91, 0.62, and 0.64, respectively. Source data for mutational signature analysis are provided in Supplementary Software 1.

Supplementary Table 1 The included cancer subtypes and patient number in CN and U.S. cohorts

23 tumor sites in ICD-10	45 Common Cancer Subtypes	CN_10,939	U.S._16,027
Lip oral cavity and pharynx _C00-C14	Nasopharyngeal Carcinoma	75	56
	Head and Neck Squamous Cell Carcinoma	94	648
Esophagus_C15	Esophageal Adenocarcinoma	12	87
	Esophageal Squamous Cell Carcinoma	914	320
Stomach_C16	Stomach Adenocarcinoma	850	497
	Intestinal and Diffuse Stomach Adenocarcinoma	123	152
Colon, Rectum, and Anus_C18-C21	Colorectal Adenocarcinoma	1541	1216
Liver _C22	Hepatocellular Carcinoma	1535	611
Gallbladder and Cholangiocarcinoma_C23-C24	Cholangiocarcinoma	859	91
	Gallbladder Carcinoma	240	32
Pancreas_C25	Pancreatic Adenocarcinoma	461	700
	Pancreatic Neuroendocrine Tumor	44	117
Traches, Bronchus and Lung_C33-C34	Lung Adenocarcinoma	1572	799
	Lung Squamous Cell Carcinoma	392	484
	Small Cell Lung Cancer	220	156
Thymus_C37	Thymic Carcinoma	33	132
Bone_C40-C41	Ewing Sarcoma	30	211
Melanoma of Skin_C43	Cutaneous Melanoma	27	618
Connective and Soft Tissue_C47+C49	Angiosarcoma	13	36
	Liposarcoma	63	48
	Leiomyosarcoma	40	91
	Nerve Sheath Tumor	15	23
	Myxofibrosarcoma	17	25
	Rhabdoid Cancer	3	69
	Rhabdomyosarcoma	44	43
	Synovial Sarcoma	36	10
	Undifferentiated Pleomorphic Sarcoma/Malignant Fibrous Histiocytoma/Spindle Cell Sarcoma	70	49
Breast_C50	Breast Invasive Carcinoma	305	1661
Cervix uteri_C53	Cervical Squamous Cell Carcinoma	76	234
Corpus uteri_C54-C55	Uterine Endometrioid Carcinoma	49	390
	Uterine Serous Carcinoma	6	106
	Uterine Clear Cell Carcinoma	2	16
Ovary_C56	Serous Ovarian Cancer	216	409
Prostate_C61	Prostate Adenocarcinoma	65	1445
Kidney and Renal Pelvis_C64-C65	Chromophobe Renal Cell Carcinoma	5	146
	Papillary Renal Cell Carcinoma	17	274
	Renal Clear Cell Carcinoma	243	472
Bladder_C66-C67	Bladder Urothelial Carcinoma	163	556
Brain and Nervous System_C70-C72	Astrocytoma	112	87
	Oligoastrocytoma	19	7
	Oligodendroglioma	53	1
	Glioblastoma	102	838
Thyroid_C73	Papillary Throid Carcinoma	71	483
Non-Hodgkin lymphoma_C82-C86; C96	Diffuse Large B-Cell Lymphoma	31	1260
Leukeamia_C91-C95	Myelogenous Leukaemia	81	321

Supplementary Table 1 Based on the 23 tumor sites (ICD-10) encompassed for the CN and U.S. comparison, 45 exact cancer subtypes were commonly shared, the corresponding total case numbers were 91.6% (10,939/11,948), and 86.2% (16,027/18,584) of all the CN and U.S. cohorts, respectively. The full list of the cancer subtypes in both cohorts are provided in Supplementary Data 1_Sequencing data_Cases and subtypes.
