# Supplementary material for: The comparison of cancer gene mutation frequencies in Chinese and U.S. patient populations
Source: Nat Commun. 2022 Sep 26;13:5651. doi: 10.1038/s41467-022-33351-4 (PMC9512793; doi:10.1038/s41467-022-33351-4)
Supplement: Supplementary file 3 — Reporting Summary [file 41467_2022_33351_MOESM3_ESM.pdf]

nature portfolio

Corresponding author(s): Prof. Zigang Dong
Last updated by author(s): Sep 7, 2022

Reporting Summary

Nature Portfolio wishes to improve the reproducibility of the work that we publish. This form provides structure for consistency and transparency in reporting. For further information on Nature Portfolio policies, see our Editorial Policies and the Editorial Policy Checklist.

Statistics

For all statistical analyses, confirm that the following items are present in the figure legend, table legend, main text, or Methods section.

n/a	Confirmed	
[ ]	[x]	The exact sample size (n) for each experimental group/condition, given as a discrete number and unit of measurement
[ ]	[x]	A statement on whether measurements were taken from distinct samples or whether the same sample was measured repeatedly
[ ]	[x]	The statistical test(s) used AND whether they are one- or two-sided<br>*Only common tests should be described solely by name; describe more complex techniques in the Methods section.*
[x]	[ ]	A description of all covariates tested
[ ]	[x]	A description of any assumptions or corrections, such as tests of normality and adjustment for multiple comparisons
[ ]	[x]	A full description of the statistical parameters including central tendency (e.g. means) or other basic estimates (e.g. regression coefficient) AND variation (e.g. standard deviation) or associated estimates of uncertainty (e.g. confidence intervals)
[ ]	[x]	For null hypothesis testing, the test statistic (e.g. F, t, r) with confidence intervals, effect sizes, degrees of freedom and P value noted<br>*Give P values as exact values whenever suitable.*
[x]	[ ]	For Bayesian analysis, information on the choice of priors and Markov chain Monte Carlo settings
[x]	[ ]	For hierarchical and complex designs, identification of the appropriate level for tests and full reporting of outcomes
[ ]	[x]	Estimates of effect sizes (e.g. Cohen's d, Pearson's r), indicating how they were calculated

Our web collection on statistics for biologists contains articles on many of the points above.

Software and code

Policy information about availability of computer code

Data collection	No software was used for data collection.

Data analysis	The GraphPad Prism.5 Version_5.01 was used to compare the mutation rates of top50 cancer genes in each of the 18 cancer types from China and U.S.<br>The Microsoft Excel Version_15.30 (170107) for Mac was used to analysis the linear correlation between different cohorts.<br>The Microsoft PowerPoint Version_15.30 (170107) for Mac was used to produce the human body image in Figure 1 .<br>The mutation proportion estimates were calculated by using the approach described by Mendiratta G, et al, 2021.<br>The SignatureAnalyzer program developed by the Getz laboratory was used to produce the mutation signatures.<br>The Wolfram Mathematica Version_13.0 was used for all the code and programs.<br>The IARC/IACR-Check (IARCcrgTools_2.05 software) was utilized to evaluate the completeness, validity, and internal consistency of the cancer epidemiological data.<br>The signatureanalyzer.spectra.get_spectra_from_maf command was used to generate the spectra files for each cancer type via using cosmic3 and the hg19.2bit reference genome assembly as the input arguments.<br>The signatureanalyzer.run_spectra command was used to generate the mutation signatures using cosmic3 and nruns=10 as the input arguments.<br>Based upon the preliminary weights, the percentage of tumors without corresponding sequencing data were calculated as value Q. The final epidemiological weights were calculated through rescaling, specifically by dividing the preliminary weights of the tumors with sequencing data by (1 - value Q).<br>The four commonly used variant callers MuTect2 (Version_4.1.0.0), VarScan2 (Version_2.4.2), SomaticSniper (Version_1.0.5.0), and MuSE (Version_1.0) Variant were used for variant aggregation and masking against the GDC TCGA Esophageal Cancer.<br>The top 50 genes within each cancer type from both populations were overlapped to determine the corresponding common rate in the top50 genes, which is calculated in 2* (TotalGeneNumber – TotalUniqueGeneNumber) / TotalGeneNumber.

For manuscripts utilizing custom algorithms or software that are central to the research but not yet described in published literature, software must be made available to editors and reviewers. We strongly encourage code deposition in a community repository (e.g. GitHub). See the Nature Portfolio guidelines for submitting code & software for further information.

Data

Policy information about availability of data

All manuscripts must include a data availability statement. This statement should provide the following information, where applicable:

- Accession codes, unique identifiers, or web links for publicly available datasets
- A description of any restrictions on data availability
- For clinical datasets or third party data, please ensure that the statement adheres to our policy

All the data used in this work are publicly available and no new data were generated in this study. The genetic mutational data are publicly available at https://www.cbioportal.org, https://dcc.icgc.org, http://www.cgga.org.cn, https://pubmed.ncbi.nlm.nih.gov/25171927/, https://pubmed.ncbi.nlm.nih.gov/32183952/, http://gdac.broadinstitute.org. The cancer epidemiological data of 2004~2016 are derived from the "China Cancer Registry Annual Report" series, which were tabulated and published by China National Cancer Center (NCC). The U.S. epidemiologically weighted cancer mutation rates, which integrated the data from SEER database and cBioPortal database are derived from the supplemental files at https://www.nature.com/articles/s41467-021-26213-y. All the output data in this study could be referred to the "Supplementary Information" and "Supplementary Data".

Field-specific reporting

Please select the one below that is the best fit for your research. If you are not sure, read the appropriate sections before making your selection.

☒ Life sciences ☐ Behavioural & social sciences ☐ Ecological, evolutionary & environmental sciences

For a reference copy of the document with all sections, see nature.com/documents/nr-reporting-summary-flat.pdf

Life sciences study design

All studies must disclose on these points even when the disclosure is negative.

Sample size	The sample size was determined as that 11,948 cancer patients with only both publicly available epidemiological information and qualified online sequencing profiles were included for analysis.
Data exclusions	Exclusion criteria were pre-established and only genes possessing protein coding mutations in the panel of 382 cancer gene census were included for analysis.
Replication	All the results and conclusions in this work are produced from analyzing of the publicly available data with regard to the cancer epidemiological data and cancer genomic sequencing data, which are not experimental findings. Neither biological replication nor technical replication are necessary for the designing and aim of this study.
Randomization	According to the "Guidelines of Chinese Cancer Registration", "Technical Protocols of Cancer Registration and Follow Up", and the standards of International Agency for Research on Cancer/International Association of Cancer Registries (IARC/IACR) on "Cancer Incidence in Five Continents, Vol. XI", cancer registries collected data on all cancers' incidence, mortality, and survival following the standards of ICD-O-3 or ICD-10, and a national criterion to evaluate the quality of Chinese cancer registration data was established following the rules of comparability, completeness, validity, and timeliness. Additionally, the genomic mutational data are collected from databases (cBioPortal, ICGC, etc.) and publications. In this process, we did not produce any new sequencing data or collect new cancer incidence data, so randomization is not relevant to this work.

Blinding

The focus of our work is integrate the cancer epidemiological data and the genetic mutation rates to produce the weighted cancer mutation frequencies in the whole cancer population in China. The cancer epidemiological data are cited from the 2004~2016 "China Cancer Registry Annual Report" series, which were published by China National Cancer Center (NCC). The genomic mutational data are collected from databases (cBioPortal, ICGC, etc.) and publications. During this process, we do not introduce any new genomic or epidemiological data in this work, so blinding is not relevant to this work in the context.

Reporting for specific materials, systems and methods

We require information from authors about some types of materials, experimental systems and methods used in many studies. Here, indicate whether each material, system or method listed is relevant to your study. If you are not sure if a list item applies to your research, read the appropriate section before selecting a response.

Materials & experimental systems

n/a	Involved in the study	
☒	☐	Antibodies
☒	☐	Eukaryotic cell lines
☒	☐	Palaeontology and archaeology
☒	☐	Animals and other organisms
☒	☐	Human research participants
☒	☐	Clinical data
☒	☐	Dual use research of concern

Methods

n/a	Involved in the study	
☒	☐	ChIP-seq
☒	☐	Flow cytometry
☒	☐	MRI-based neuroimaging
