# Supplementary material for: The comparison of cancer gene mutation frequencies in Chinese and U.S. patient populations
Source: Nat Commun. 2022 Sep 26;13:5651. doi: 10.1038/s41467-022-33351-4 (PMC9512793; doi:10.1038/s41467-022-33351-4)
Supplement: Supplementary file 4 — Description of Additional Supplementary Files [file 41467_2022_33351_MOESM4_ESM.docx]

**Description of Additional Supplementary Files**

File Name: Supplementary Data 1

Description: Sequencing data

File Name: Supplementary Data 2

Description: Treatment status data

File Name: Supplementary Data 3

Description: Epidemiological data

File Name: Supplementary Data 4

Description: Variant callers data

File Name: Supplementary Data 5

Description: Statistics

File Name: Supplementary Software 1

Description: Code data
