# Supplementary material for: The comparison of cancer gene mutation frequencies in Chinese and U.S. patient populations
Source: Nat Commun. 2022 Sep 26;13:5651. doi: 10.1038/s41467-022-33351-4 (PMC9512793; doi:10.1038/s41467-022-33351-4)
Supplement: Supplementary file 9 — Supplementary Software 1 [file 41467_2022_33351_MOESM9_ESM.zip › Supplementary Software 1 New/Analysis_Code.pdf]

Epidemiological Data Formatting

In order to facilitate comparisons between the Chinese and US patient populations, we first normalize the epidemiological datasets for each country. The 23 cancer types with available sequencing data (for both populations) are listed in “/Supplementary Software 1/Other/tumorSitesInICD10.csv”. The “epidemiologicalDataUS.txt” (originally called “Genomics_Output_Processed.txt”) file is derived from the Supplementary Software 1 directory of

Mendiratta, G., Ke, E., Aziz, M. *et al*. Cancer gene mutation frequencies for the U.S. population. *Nat Commun* **12,** 5961 (2021). https://doi.org/10.1038/s41467-021-26213-y.

Data usage is licensed under the Creative Commons Attribution 4.0 International License (http://creativecommons.org/licenses/by/4.0/). As the epidemiological data contains more cancer types than those which are represented in the sequencing data, those cancers represented in the epidemiological data but not in the sequencing data are removed and the remaining epidemiological data is rescaled. This process is executed below below:

Data Formatting (US)

```
usWeights = Import[
   "/path/to/Supplementary Software 1/Input Files/epidemiologicalDataUS.txt", "TSV"];
```

```
unmodWeightsUS =
  Partition[Riffle[usWeights[[3 ;; 45, 1]], Table[Total[Drop[usWeights[[i]], 2]], {i, 3, 45}] /
     Total[Table[Total[Drop[usWeights[[i]], 2]], {i, 3, 45}]]], 2]
```

The indices of the unused tissue types are contained within the curly braces ({}) below. Together, they comprise ~6.64603% of the total available data.

```
Total[unmodWeightsUS[[{4, 7, 11, 12, 13, 18, 23, 24, 26, 27, 30, 31, 35, 37, 43}]][[All, 2]]]
```

Once these unused values are removed, the data is rescaled by dividing the remaining values by .9335 (1-0.0664603).

```
Delete[unmodWeightsUS, {{4}, {7}, {11}, {12}, {13}, {18},
    {23}, {24}, {26}, {27}, {30}, {31}, {35}, {37}, {43}}][[All, 2]] / 0.9335
```

The original labels are then riffled and the cells are partitioned such that the weights of each tissue are group with their respective tissue types:

```
Partition[Riffle[Delete[unmodWeightsUS,
    {{4}, {7}, {11}, {12}, {13}, {18}, {23}, {24}, {26}, {27}, {30}, {31}, {35}, {37}, {43}}][[All, 1]],
  Delete[unmodWeightsUS, {{4}, {7}, {11}, {12}, {13}, {18}, {23}, {24},
      {26}, {27}, {30}, {31}, {35}, {37}, {43}}][[All, 2]] / 0.9335], 2]
```

```
Export["/path/to/Destination/prelimEpidemiologicalWeightsUS.txt",
 Partition[Riffle[Delete[unmodWeightsUS,
     {{4}, {7}, {11}, {12}, {13}, {18}, {23}, {24}, {26}, {27}, {30}, {31}, {35}, {37}, {43}}][[
    All, 1]], Delete[unmodWeightsUS, {{4}, {7}, {11}, {12}, {13}, {18}, {23},
       {24}, {26}, {27}, {30}, {31}, {35}, {37}, {43}}][[All, 2]] / 0.9335], 2]]
```

Colon/Rectum types are combined (added) to yield the “Colorectum” type. ALL/CLL/Other Leukemia/AML/CML are combined (added) to yield the “Leukemia” type. This step performed in an Excel spreadsheet for the sake of convenience. After making the modifications, the final version of the file was saved as “epidemiologicalWeightsUS_final.txt”. Both files are presented in the Results directory.

Data Formatting (CN)

Below we take the same factors into consideration follow a similar process to normalize the Chinese epidemiological data:

```
cnWeights =
  Import["/path/to/Supplementary Software 1/Input Files/epidemiologicalDataCN.xlsx",
    "XLSX"][[1]];
```

After importing the Chinese epidemiological data, the rows & columns of interest are retained:

```
cnWeights = cnWeights[[1 ;; 60, 1 ;; 16]];
```

The percentage that each tissue type type contributes to the whole is calculated:

```
newestPercentagesCN = Rest[cnWeights[[All, 16]]] / Total[Rest[cnWeights[[All, 16]]]] * 100
```

The original labels are then riffled and the cells are partitioned such that the weights of each tissue are group with their respective tissue types:

```
Partition[Riffle[Rest[cnWeights[[All, 2]]], newestPercentagesCN], 2]
```

The indices of the unused tissue types are contained within the curly braces ({}) below. Together, they comprise ~5.3709% of the total available data.

```
cnPercToRetain =
 Delete[Partition[Riffle[Rest[cnWeights[[All, 2]]], newestPercentagesCN], 2],
  {{12}, {19}, {20}, {25}, {26}, {27}, {30}, {31}, {36}, {37},
   {38}, {40}, {41}, {46}, {47}, {50}, {51}, {52}, {54}, {55}, {59}}]
```

```
Total[cnPercToRetain[[All, 2]]]
```

Once these unused values are removed, the data is rescaled by dividing the remaining values by 0.946291 (1-0.053709). The original labels are then riffled and the cells are partitioned such that the weights of each tissue are group with their respective tissue types:

```
Partition[Riffle[cnPercToRetain[[All, 1]], (cnPercToRetain[[All, 2]] / 0.946291)], 2]
```

```
Export["/path/to/Destination/prelimEpidemiologicalWeightsCN.txt",
 Partition[Riffle[cnPercToRetain[[All, 1]], (cnPercToRetain[[All, 2]] / 0.946291)], 2]]
```

Lip/Tongue/Mouth/Salivary Glands/Tonsil/Other Oropharynx/Nasopharynx/Hypopharynx/Pharynx Unspecified types are combined (added) to yield the “Lip oral cavity and pharynx” type. Colon/Rectum/Anus types are combined (added) to yield the “Colon, Rectum, and Anus” type. Liver type was renamed “Liver and intrahepatic bile ducts”. Gall bladder etc. was renamed “Gallbladder and Cholangiocarcinoma”. Other Thoracic Organs was renamed “Thymus”. Corpus Uteri/Uterus Unspecified are combined (added) to yield the “Corpus uteri” type. Proslate was renamed (corrected) “Prostate”. Kidney/Renal Pelvis are combined (added) to yield the “Kidney and Renal Pelvis” type. Ureter/Bladder are combined (added) to yield the “Bladder” type. Brain, Nervous System was renamed “Brain and Nervous System”. Thyroid Gland was renamed “Thyroid”. Lymphoid Leukaemia/Myeloid Leukaemia/Leukaemia Unspecified are combined (added) to yield the “Leukaemia” type. This step was done in an Excel spreadsheet for the sake of convenience. After making the modifications, the final version of the file was saved as “epidemiologicalWeightsCN_final.txt”. Both files are presented in the Results directory.

Mutation Data Formatting

Data Formatting and Processing (US)

The original US sequencing data file provided in the Supplementary Software 1 directory of Mendiratta, G., Ke, E., Aziz, M. *et al*. Cancer gene mutation frequencies for the U.S. population. *Nat Commun* **12,** 5961 (2021). https://doi.org/10.1038/s41467-021-26213-y (originally called “Genomics_Output_processed.txt”; the name was changed to “mutationCountsUS.txt” for use in this notebook) organizes tumor tissues into fine-grained categories. To facilitate comparison with Chinese sequencing data, mutation counts within specific fine-grained tumor tissue categories were combined to yield coarse-grained tissue-specific categories. The coarse-grained re-mapping scheme was based upon the spreadsheet provided in Supplementary Data 2 of the Mendiratta, G. *et al*. and the “Input_Seer_Histology_Recode_Map.txt” file located in the SEER_Analysis directory of their Supplementary Software . The first row of the “mutationCountsUS.txt” file indicates the Rosetta classification in which all mutations within that respective column were observed; this classification was used to add mutations observed across individual classifications into specific tissue groupings. The files can be found in “/Supplementary Software 1/Others/MendirattaEtAl_SuppData2” and “/Supplementary Software 1/Others/MendirattaEtAl_Input_SEER_Histology_Recode_Map.txt”. The “Genomics_Output_Processed.txt” file is also derived from the Mendiratta, G. *et al*. Supplementary Software 1 directory. These data that were not generated by us are licensed under the Creative Commons Attribution 4.0 International License (http://creativecommons.org/licenses/by/4.0/). The “mutationCountsUS.txt” data is first imported and the columns corresponding to each tissue type (with associated sequencing data) was retained:

```
mutFileUS =
  Import["/path/to/Supplementary Software 1/Input Files/mutationCountsUS.txt", "TSV"];
```

```
mutListUS = Join[{mutFileUS⟦All, 1⟧},
  {Prepend[Rest[mutFileUS⟦All, 24⟧] + Rest[mutFileUS⟦All, 19⟧], "Lip/Oral"]},
  {Prepend[Rest[mutFileUS⟦All, 20⟧] + Rest[mutFileUS⟦All, 31⟧], "Esophagus"]},
  {Prepend[Rest[mutFileUS⟦All, 32⟧], "Stomach"]},
  {Prepend[Rest[mutFileUS⟦All, 34⟧], "Colorectum"]},
  {Prepend[Rest[mutFileUS⟦All, 17⟧], "Hepatic"]},
  {Prepend[Rest[mutFileUS⟦All, 35⟧] + Rest[mutFileUS⟦All, 44⟧], "Gallbladder"]},
  {Prepend[Rest[mutFileUS⟦All, 37⟧] + Rest[mutFileUS⟦All, 36⟧] + Rest[mutFileUS⟦All, 13⟧],
    "Pancreas"]}, {Prepend[Rest[mutFileUS⟦All, 33⟧] +
     Rest[mutFileUS⟦All, 22⟧] + Rest[mutFileUS⟦All, 14⟧], "Lung"]},
  {Prepend[Rest[mutFileUS⟦All, 58⟧], "Bone"]},
  {Prepend[Rest[mutFileUS⟦All, 5⟧] + Rest[mutFileUS⟦All, 42⟧] + Rest[mutFileUS⟦All, 4⟧] +
     Rest[mutFileUS⟦All, 63⟧] + Rest[mutFileUS⟦All, 43⟧] + Rest[mutFileUS⟦All, 41⟧] +
     Rest[mutFileUS⟦All, 38⟧] + Rest[mutFileUS⟦All, 57⟧] + Rest[mutFileUS⟦All, 50⟧],
    "Soft Tissue"]}, {Prepend[Rest[mutFileUS⟦All, 12⟧] +
     Rest[mutFileUS⟦All, 47⟧] + Rest[mutFileUS⟦All, 11⟧], "Melanoma"]},
  {Prepend[Rest[mutFileUS⟦All, 29⟧] + Rest[mutFileUS⟦All, 51⟧], "Breast"]},
  {Prepend[Rest[mutFileUS⟦All, 21⟧] + Rest[mutFileUS⟦All, 28⟧], "Cervix"]},
  {Prepend[Rest[mutFileUS⟦All, 27⟧] + Rest[mutFileUS⟦All, 48⟧], "Corpus Uteri"]},
  {Prepend[Rest[mutFileUS⟦All, 26⟧], "Ovary"]},
  {Prepend[Rest[mutFileUS⟦All, 30⟧], "Prostate"]},
  {Prepend[Rest[mutFileUS⟦All, 16⟧], "Bladder"]},
  {Prepend[Rest[mutFileUS⟦All, 25⟧] +
     Rest[mutFileUS⟦All, 45⟧] + Rest[mutFileUS⟦All, 49⟧], "Kidney"]},
  {Prepend[Rest[mutFileUS⟦All, 61⟧] + Rest[mutFileUS⟦All, 7⟧] +
     Rest[mutFileUS⟦All, 59⟧] + Rest[mutFileUS⟦All, 10⟧] + Rest[mutFileUS⟦All, 9⟧] +
     Rest[mutFileUS⟦All, 62⟧] + Rest[mutFileUS⟦All, 60⟧], "Brain/Nervous"]},
  {Prepend[Rest[mutFileUS⟦All, 18⟧], "Thyroid"]},
  {Prepend[Rest[mutFileUS⟦All, 15⟧], "Thymus"]},
  {Prepend[Rest[mutFileUS⟦All, 66⟧] + Rest[mutFileUS⟦All, 68⟧] +
     Rest[mutFileUS⟦All, 67⟧] + Rest[mutFileUS⟦All, 69⟧] + Rest[mutFileUS⟦All, 65⟧] +
     Rest[mutFileUS⟦All, 64⟧] + Rest[mutFileUS⟦All, 40⟧], "NHL"]},
  {Prepend[Rest[mutFileUS⟦All, 39⟧] + Rest[mutFileUS⟦All, 72⟧] + Rest[mutFileUS⟦All, 73⟧] +
     Rest[mutFileUS⟦All, 71⟧] + Rest[mutFileUS⟦All, 74⟧], "Leukemia"]}];
```

The patient number within each tissue cohort is found in the last row of each column (indexed with -1):

```
forCondProbUS = Rest@mutListUS⟦All, -1⟧
```

Important: Note that the row/column pairs are standardized in mutList and in the imported epidemiological weights below:

```
weightVecUS = Import[
  "/path/to/Supplementary Software 1/Input Files/epidemiologicalWeightsUS_final.csv",
  "CSV"]
```

Weighted percentages are calculated below. The contents of the listPercentages variable show the mutated frequency of all genes:

```
mutListUS = Transpose[mutListUS];
```

```
listPercentagesUS = {};
Do[(
  AppendTo[listPercentagesUS, {mutListUS[[i, 1]],
    Total[(Rest[mutListUS[[i]]] / forCondProbUS) * weightVecUS[[All, 2]] * 100}]
 ), {i, 2, Length@mutListUS - 1}]
```

Total percentages sorted in descending order:

```
fullGeneUnsortedUS = ReverseSortBy[listPercentagesUS, Last]
```

Since we are concerned with the cancer census genes, we use the Position function to highlight only those genes within the cancer census panel. The data can then be exported to Excel (or visualized using Mathematica if one prefers):

```
goisUS = DeleteCases[Rest[
    Import["/path/to/Supplementary Software 1/Input Files/cancerGenePanelUS.xlsx",
      "XLSX"][[1]][[All, 2]]], ""];
```

```
ReverseSortBy[
 fullGeneUnsortedUS[[Flatten[Position[fullGeneUnsortedUS[[All, 1]], #] & /@ goisUS]]], Last]
```

```
Export["/path/to/Destination/weightedMutationPercentagesUS.csv",
 ReverseSortBy[fullGeneUnsortedUS[[
   Flatten[Position[fullGeneUnsortedUS[[All, 1]], #] & /@ goisUS]]], Last]]
```

The Poisson distributed US dataset used for the simulation is generated below:

```
simValuesUS = {};
Do[(
  pos = Position[mutListUS[[All, 1]], goisUS[[i]]];
  zerosVec = Rest[mutListUS[[Flatten[pos][[1]]]]];
  poissonTable = {};
  Do[
   (
    If[SameQ[zerosVec[[k]], 0],
      AppendTo[poissonTable, Table[0, 2000]], AppendTo[poissonTable,
       RandomVariate[PoissonDistribution[zerosVec[[k]] / forCondProbUS[[k]]], 2000]]];
   ),
   {k, 1, 23}];
  listSim = {};
  Do[(
    AppendTo[listSim, (Total[poissonTable[[All, l]] * weightVecUS[[All, 2]]])];
   ), {l, 1, 2000}];
  AppendTo[simValuesUS,
   {goisUS[[i]], Mean[listSim] * 100, N[1.96 * (StandardDeviation[listSim] / Sqrt[2000])] * 100}]
 ), {i, 1, Length@goisUS}]
```

```
ReverseSortBy[simValuesUS, #[[2]] &]
```

The data can then be exported to Excel (or visualized using Mathematica if one prefers):

```
Export["/path/to/Destination/simulatedMutationPercentagesUS.csv",
 ReverseSortBy[simValuesUS, #[[2]] &]]
```

Data Formatting and Processing (CN)

Below we take the same factors into consideration follow a similar process to generate the weighted percentages and simulated dataset for the Chinese data:

```
mutListCN =
  Import["/path/to/Supplementary Software 1/Input Files/mutationCountsCN.csv", "CSV"];
```

```
weightVecCN = Import["/path/to/Supplementary Software
     1/Input Files/epidemiologicalWeightsCN_final.csv", "CSV"];
```

```
forCondProbCN = weightVecCN[[All, 2]]
```

```
weightVecCN = weightVecCN[[All, 4]]
```

Since we are concerned with the cancer census genes, we use the Position function to highlight only those genes within the cancer census panel. The data can then be exported to Excel (or visualized using Mathematica if one prefers):

```
goisCN = DeleteCases[Rest[
    Import["/path/to/Supplementary Software 1/Input Files/cancerGenePanelCN.xlsx",
      "XLSX"][[1]][[All, 2]]], ""];
```

```
listPercentagesCN = {};
Do[(
  zerosVec = Table[0, 23];
  pos = Position[mutListCN, goisCN[[i]]];
  Do[(
    zerosVec[[(pos[[j, 2]] + 2) / 3]] = mutListCN[[pos[[j, 1]], pos[[j, 2]] + 1]]
   ), {j, 1, Length@pos}];
  AppendTo[listPercentagesCN, Total[(zerosVec / forCondProbCN) * weightVecCN]]
 ), {i, 1, Length@goisCN}]
```

```
fullGeneSortedCN = ReverseSortBy[Partition[Riffle[goisCN, listPercentagesCN], 2], Last]
```

The data can then be exported to Excel (or visualized using Mathematica if one prefers):

```
Export["/path/to/Destination/weightedMutationPercentagesCN.csv", fullGeneSortedCN]
```

The Poisson distributed CN dataset used for the simulation is generated below:

```
simValuesCN = {};
Do[(
  zerosVec = Table[0, 23];
  pos = Position[mutListCN, goisCN[[i]]];
  Do[(
    zerosVec[[(pos[[j, 2]] + 2) / 3]] = mutListCN[[pos[[j, 1]], pos[[j, 2]] + 1]]
   ), {j, 1, Length@pos}];
  poissonTable = {};
  Do[
   (
    If[SameQ[zerosVec[[k]], 0],
      AppendTo[poissonTable, Table[0, 2000]], AppendTo[poissonTable,
       RandomVariate[PoissonDistribution[zerosVec[[k]] / forCondProbCN[[k]]], 2000]]];
   ),
   {k, 1, 23}];
  listSim = {};
  Do[(
    AppendTo[listSim, (Total[poissonTable[[All, l]] * weightVecCN])];
   ), {l, 1, 2000}];
  AppendTo[simValuesCN,
   {goisCN[[i]], Mean[listSim], N[1.96 * (StandardDeviation[listSim] / Sqrt[2000])]}]
 ), {i, 1, Length@goisCN}]
```

The data can then be exported to Excel (or visualized using Mathematica if one prefers):

```
Export["/path/to/Destination/simulatedMutationPercentagesCN.csv",
 ReverseSortBy[simValuesCN, #[[2]] &]]
```

There are slight differences in gene names between the Chinese and US datasets. The following gene names in output for the Chinese weighted mutation and simulated dataset were manually changed in Excel for the sake of convenience: WISP3 -> CCN6; H3F3A -> H3-3A; MRE11A -> MRE11; WHSC1 -> NSD2; PARK2 -> PRKN. Within the output for the US weighted mutation and simulated dataset, C11ORF30 was manually changed to C11orf30.

Partitioning MSK Data Based on Smoking Status and Gender

The next section is aimed at partitioning the MSK data, with emphasis placed on the LUAD cohort, with respect to smoking and treatment status. This is because there is significant ambiguity in the patient clinical information with regards to smoking history between the 2017 and 2020 data, so only those patients whose smoking status was the same in both clinical data files were retained. The scripts below set up a truth vector designed to flag ambiguous smokers in the MSK data. The intersection of MSK LUAD patients and those with unambiguous smoking status is then retained and used for subsequent analyses.

Patient and sample clinical data are first imported:

```
samp2017 = Import[
   "/path/to/Supplementary Software 1/Input Files/msk2017_ClinicalSample.txt", "TSV"];

samp2020 = Import[
   "/path/to/Supplementary Software 1/Input Files/msk2020_ClinicalSample.txt", "TSV"];

clin17 =
  Import["/path/to/Supplementary Software 1/Input Files/msk2017_ClinicalPatient.txt",
   "TSV"];

clin20 =
  Import["/path/to/Supplementary Software 1/Input Files/msk2020_ClinicalPatient.txt",
   "TSV"];
```

Non-smokers, smokers (current and former), and those with unknown smoking history are identified using the patient clinical data for 2017 and 2020:

```
In[ ]:= neverSmokers2017 = clin17[[Flatten@Position[clin17[[All, 4]], "Never"], {1, 2, 4}]];

In[ ]:= unknown2017 = clin17[[Flatten@Position[clin17[[All, 4]], "Unknown"], {1, 2, 4}]];

In[ ]:= Table[unknown2017[[i, 3]] = "NA", {i, 1, Length@unknown2017}];

In[ ]:= smokers2017 = clin17[[Flatten@Position[clin17[[All, 4]], "Prev/Curr Smoker"], {1, 2, 4}]];

In[ ]:= neverSmokers2020 = clin20[[Flatten@Position[clin20[[All, 5]], "Never smoker"]]];
```

```
In[ ]:= Table[neverSmokers2020[[i, 3]] = "Never", {i, 1, Length@neverSmokers2020}];

In[ ]:= unknown2020 = clin20[[Flatten@Position[clin20[[All, 5]], "NA"]]];

In[ ]:= smokers2020 =
          Join[clin20[[Flatten@Position[clin20[[All, 5]], "Former Smoker"], {1, 2, 5, 7}]],
           clin20[[Flatten@Position[clin20[[All, 5]], "Current Smoker"], {1, 2, 5, 7}]]];

In[ ]:= Table[smokers2020[[i, 3]] = "Prev/Curr Smoker", {i, 1, Length@smokers2020}];
```

Patient names are compiled into a vector:

```
In[ ]:= comPatList =
          Union[Join[neverSmokers2017[[All, 1]], unknown2017[[All, 1]], smokers2017[[All, 1]],
            neverSmokers2020[[All, 1]], unknown2020[[All, 1]], smokers2020[[All, 1]]]];
```

Smoking status for each patient across 2017 and 2020 is encoded into a truth vector:

```
truthVec = {MemberQ[neverSmokers2017[[All, 1]], #], MemberQ[neverSmokers2020[[All, 1]], #],
     MemberQ[unknown2017[[All, 1]], #], MemberQ[unknown2020[[All, 1]], #],
     MemberQ[smokers2017[[All, 1]], #], MemberQ[smokers2020[[All, 1]], #]} & /@ comPatList;
```

Based on the truth vector, unambiguous non-smokers are encoded by:
{True, True, False, False,False,False}, {True, False, False, False,False,False}, and {False, True, False, False, False, False}.
Based on the truth vector, unambiguous smokers are encoded by:
{False, False, False, False, True, True}, {False, False, False, False, True, False}, and {False, False, False, False, False, True}.

```
unambigNever =
  comPatList[[Flatten[Join[Position[truthVec, {True, True, False, False, False, False}],
     Position[truthVec, {True, False, False, False, False, False}],
     Position[truthVec, {False, True, False, False, False, False}]]]]];

unambigSmoker =
  comPatList[[Flatten[Join[Position[truthVec, {False, False, False, False, True, True}],
     Position[truthVec, {False, False, False, False, True, False}],
     Position[truthVec, {False, False, False, False, False, True}]]]]];
```

Using the clinical sample data, one can focus on specific tissues of interest by identifying members belonging to each cohort. Subtypes within each tissue cohort can also be identified:

```
In[ ]:= poi17 =
          samp2017[[Flatten[Position[samp2017[[All, 15]], #] & /@ Union[Drop[samp2017[[All, 15]], 5]][[
              {7, 9, 12, 14, 16, 17, 22, 23, 24, 27, 29, 31, 37, 38, 39, 44, 45, 52, 55}]]]]];

In[ ]:= Union[Drop[samp2017[[All, 15]], 5]][[
         {7, 9, 12, 14, 16, 17, 22, 23, 24, 27, 29, 31, 37, 38, 39, 44, 45, 52, 55}]]
```

```
In[ ]:= final17 = poi17[[Flatten[Position[poi17[[All, 16]], #] & /@
          (Union[poi17[[All, 16]]])[[{19, 21, 22, 79, 27, 158, 40, 174, 176, 179, 44, 48, 50, 76,
            163, 60, 83, 109, 52, 57, 68, 69, 78, 93, 41, 80, 85, 86, 89, 36, 46, 72, 54,
            104, 151, 6, 77, 120, 161, 135, 32, 37, 123, 141, 164, 166, 160, 56, 95, 124}]]]];

In[ ]:= final20 = samp2020[[Flatten[Position[samp2020[[All, 5]], #] & /@
          (Union@samp2020[[Flatten[Position[samp2020[[All, 4]], #] & /@ (Union[Drop[samp2020[[
                        All, 4]], 5]])[[{6, 8, 11, 14, 16, 24, 37, 38}]]]][[All, 5]])[[{3, 4, 5, 6, 8}]]]];

In[ ]:= subtypes17 = Union@final17[[Flatten[Position[final17[[All, 15]], #] & /@
            Union[final17[[All, 15]]][[{2, 4, 6, 9, 12}]]]]][[All, 16]];
```

As we are interested in Lung Cancer, we single this cohort out from the group.

```
In[ ]:= five17Lung =
         final17[[Flatten[Position[final17[[All, 16]], #] & /@ subtypes17[[{15, 16, 17, 18}]]]]];

In[ ]:= subtypes20 = Union@final20[[
          Flatten[Position[final20[[All, 5]], #] & /@ Union[final20[[All, 5]]][[{2, 3, 4}]]]]][[All, 5]];

In[ ]:= patListLung = DeleteDuplicates[five17Lung[[All, 1]]];
```

LUAD patients are further grouped out of the patListLung (total lung patient group) based on smoking status unambiguity:

```
In[ ]:= patListLUAD =
         Intersection[Join[unambigNever, unambigSmoker], DeleteDuplicates[five17Lung[[
             Flatten[Position[five17Lung[[All, 16]], "Lung Adenocarcinoma"]]]][[All, 1]]]];
```

The mutation files for the 2017 and 2020 MSK datasets are imported and a small modification is made to format the Tumor_Sample_Barcode for convenient indexing:

```
mutationFile1 = Import[
   "/path/to/Supplementary Software 1/Input Files/msk2017_MutationsExtended.txt",
   "TSV"];

In[ ]:= mutationFile1 = mutationFile1[[Flatten[Position[mutationFile1[[All, 9]], #] & /@ Delete[
            Union[Rest@mutationFile1[[All, 9]]], {{1}, {2}, {5}, {15}, {26}, {27}, {39}, {55}, {56}}]]]];

In[ ]:= Do[(mutationFile1[[i, 17]] = StringDrop[mutationFile1[[i, 17]], -8]),
         {i, 2, Length@mutationFile1}];

mutationFile2 = Import[
   "/path/to/Supplementary Software 1/Input Files/msk2020_MutationsExtended.txt",
   "TSV"];

In[ ]:= mutationFile2 = mutationFile2[[Flatten[Position[mutationFile2[[All, 9]], #] & /@
          Delete[Union[Rest[mutationFile2[[All, 9]]]], {{1}, {2}, {3}, {9}, {30}, {31}, {32}}]]]];

In[ ]:= Do[(mutationFile2[[i, 17]] = StringDrop[mutationFile2[[i, 17]], -4]),
         {i, 2, Length@mutationFile2}];
```

Partitioning the MSK LUAD mutations for the unambiguous smokers and non-smokers based on gender. The script iteratively updates the mutIndex variable, which is initially set as {0,0,1}, using patient clinical information.

The first value of the mutIndex variable encodes for gender (a 0 value encodes for a male, a 1 value encodes for a female).

The second value of the mutIndex variable encodes for smoking status (a 0 value encodes for a non-smoker, a 1 value encodes for a smoker, and “x” value encodes for an unknown smoker).

The final value of the mutIndex variable encodes which year the mutation data is derived from (a 0 value encodes for 2017, a 1 value encodes for 2020). By default, the most recent mutation data is used for analysis.

Examples:

A mutIndex value of {1, 0, 1} encodes for a female non-smoker LUAD patient whose mutation data is available for 2020.

A mutIndex value of {0,1,0} encodes for a male smoker LUAD patient whose mutation data is available for 2017.

```
In[ ]:= luadNsM = {};
luadNsF = {};
luadSM = {};
luadSF = {};
Do[(
  pos = Position[mutationFile2[[All, 17]], patListLUAD[[i]]];
  pos1 = Position[clin20[[All, 1]], patListLUAD[[i]]];
  mutIndex = {0, 0, 1};
  If[SameQ[pos, {}], (pos = Position[mutationFile1[[All, 17]], patListLUAD[[i]]];
    pos1 = Position[clin17[[All, 1]], patListLUAD[[i]]]; mutIndex = {0, 0, 0};)];
  If[SameQ[mutIndex, {0, 0, 1}],
   (If[SameQ[clin20[[pos1[[1]], 2]][[1]], "Male"], mutIndex[[1]] = 0;, mutIndex[[1]] = 1;];
    If[SameQ[clin20[[pos1[[1]], 5]][[1]], "Current Smoker"] ||
      SameQ[clin20[[pos1[[1]], 5]][[1]], "Former Smoker"], mutIndex[[2]] = 1];
    If[SameQ[clin20[[pos1[[1]], 5]][[1]], "Never smoker"], mutIndex[[2]] = 0;];
    If[SameQ[clin20[[pos1[[1]], 5]][[1]], "NA"], mutIndex[[2]] = "x";])];
  If[SameQ[mutIndex, {0, 0, 0}],
   (If[SameQ[clin17[[pos1[[1]], 2]][[1]], "Male"], mutIndex[[1]] = 0;, mutIndex[[1]] = 1;];
    If[SameQ[clin17[[pos1[[1]], 4]][[1]], "Prev/Curr Smoker"], mutIndex[[2]] = 1];
    If[SameQ[clin17[[pos1[[1]], 4]][[1]], "Never"], mutIndex[[2]] = 0;];
    If[SameQ[clin17[[pos1[[1]], 4]][[1]], "Unknown"], mutIndex[[2]] = "x";])];
  If[SameQ[{0, 0, 0}, mutIndex], (AppendTo[luadNsM, mutationFile1[[Flatten@pos]]];)];
  If[SameQ[{0, 0, 1}, mutIndex], (AppendTo[luadNsM, mutationFile2[[Flatten@pos]]];)];
  If[SameQ[{1, 0, 0}, mutIndex], (AppendTo[luadNsF, mutationFile1[[Flatten@pos]]];)];
  If[SameQ[{1, 0, 1}, mutIndex], (AppendTo[luadNsF, mutationFile2[[Flatten@pos]]];)];
  If[SameQ[{0, 1, 0}, mutIndex], (AppendTo[luadSM, mutationFile1[[Flatten@pos]]];)];
  If[SameQ[{0, 1, 1}, mutIndex], (AppendTo[luadSM, mutationFile2[[Flatten@pos]]];)];
  If[SameQ[{1, 1, 0}, mutIndex], (AppendTo[luadSF, mutationFile1[[Flatten@pos]]];)];
  If[SameQ[{1, 1, 1}, mutIndex], (AppendTo[luadSF, mutationFile2[[Flatten@pos]]];)];
 ), {i, 1, Length@patListLUAD}]
```

Below, the mutations for each gender/smoking grouping are obtained and subsequently filtered using the cancer gene census panel. As indicated within the manuscript, multiple mutations within each single gene are only counted once (this is ensured using the Union function, which identifies unique elements within a list). NsF denotes Non-smoking Females, NsM denotes Non-smoking Males, SM denotes Smoking Males, and SF denotes Smoking Females.

In[ ]:=
```
luadNsF = Flatten[luadNsF, 1];
uniquePatientsNsF = DeleteDuplicates@luadNsF[[All, 17]];
luadNsFMutList = {};
Do[(
   pos = Position[luadNsF[[All, 17]], uniquePatientsNsF[[i]]];
   AppendTo[luadNsFMutList, Union@luadNsF[[Flatten@pos]][[All, 1]]];
  ), {i, 1, Length@uniquePatientsNsF}]
```

In[ ]:=
```
Length@uniquePatientsNsF
```

In[ ]:=
```
talliedNsFLUADMutList = ReverseSortBy[Tally[Flatten[luadNsFMutList]], Last];

Export["/path/to/Destination/mskFemaleNonSmokerMutationCounts.csv",
 talliedNsFLUADMutList[[
  Flatten@Position[MemberQ[goisUS, #] & /@ talliedNsFLUADMutList[[All, 1]], True]]]]
```

In[ ]:=
```
luadSF = Flatten[luadSF, 1];
uniquePatientsSF = DeleteDuplicates@luadSF[[All, 17]];
luadSFMutList = {};
Do[(
   pos = Position[luadSF[[All, 17]], uniquePatientsSF[[i]]];
   AppendTo[luadSFMutList, Union@luadSF[[Flatten@pos]][[All, 1]]];
  ), {i, 1, Length@uniquePatientsSF}]
```

In[ ]:=
```
Length@uniquePatientsSF
```

In[ ]:=
```
talliedSFLUADMutList = ReverseSortBy[Tally[Flatten[luadSFMutList]], Last];

Export["/path/to/Destination/mskFemaleSmokerMutationCounts.csv",
 talliedSFLUADMutList[[
  Flatten@Position[MemberQ[goisUS, #] & /@ talliedSFLUADMutList[[All, 1]], True]]]]
```

In[ ]:=
```
luadNsM = Flatten[luadNsM, 1];
uniquePatientsNsM = DeleteDuplicates@luadNsM[[All, 17]];
luadNsMMutList = {};
Do[(
   pos = Position[luadNsM[[All, 17]], uniquePatientsNsM[[i]]];
   AppendTo[luadNsMMutList, Union@luadNsM[[Flatten@pos]][[All, 1]]];
  ), {i, 1, Length@uniquePatientsNsM}]
```

In[ ]:=
```
Length@uniquePatientsNsM
```

In[ ]:=
```
talliedNsMLUADMutList = ReverseSortBy[Tally[Flatten[luadNsMMutList]], Last];

Export["/path/to/Destination/mskMaleNonSmokerMutationCounts.csv",
 talliedNsMLUADMutList[[
  Flatten@Position[MemberQ[goisUS, #] & /@ talliedNsMLUADMutList[[All, 1]], True]]]]
```

```
In[ ]:= luadSM = Flatten[luadSM, 1];
uniquePatientsSM = DeleteDuplicates@luadSM[[All, 17]];
luadSMMutList = {};
Do[(
   pos = Position[luadSM[[All, 17]], uniquePatientsSM[[i]]];
   AppendTo[luadSMMutList, Union@luadSM[[Flatten@pos]][[All, 1]]];
  ), {i, 1, Length@uniquePatientsSM}]
```

```
In[ ]:= Length@uniquePatientsSM
```

```
In[ ]:= talliedSMLUADMutList = ReverseSortBy[Tally[Flatten[luadSMMutList]], Last];

Export["/path/to/Destination/mskMaleSmokerMutationCounts.csv", talliedSMLUADMutList[[
   Flatten@Position[MemberQ[goisUS, #] & /@ talliedSMLUADMutList[[All, 1]], True]]]]

totLUADMutations = ReverseSortBy[Tally[Flatten[
      Join[luadNsFMutList, luadNsMMutList, luadSFMutList, luadSMMutList]]], Last];

Export["/path/to/Destination/mskTotalMutationsLUAD.csv", totLUADMutations[[
   Flatten[Position[MemberQ[goisUS, #] & /@ totLUADMutations[[All, 1]], True]]]]]
```

Formatting Data for Mutation Signature Analysis

MSK Female Untreated LUAD Non-smoker Patients

It should be noted that treatment status is only available in the 2020 MSK data. Untreated female LUAD patients from the MSK data are identified below. Once treatment status is identified, one can index back into the file generated previously to get the SNPs for mutation signature analysis. The clinical information is first obtained for all non-smoking females:

```
In[ ]:= clinNsF = clin20[[Flatten[Position[clin20[[All, 1]], #] & /@ uniquePatientsNsF]]];
```

Untreated non-smoking females are then identified:

```
In[ ]:= clinNsF[[Flatten@Position[clinNsF[[All, 7]], "Untreated"]]][[All, 1]]
```

Total mutations for untreated non-smoking females are identified:

```
untreatedMutNsF = luadNsF[[Flatten[Position[luadNsF[[All, 17]], #] & /@
      clinNsF[[Flatten@Position[clinNsF[[All, 7]], "Untreated"]]][[All, 1]]]]];
```

The SNP mutations are then obtained from the total mutations of the non-smoking females. The SNPs from the top 50 mutated genes are then obtained:

```
untreatedSNPNsF = untreatedMutNsF[[
    Flatten@Position[untreatedMutNsF[[All, 11]], "SNP"], {1, 17, 5, 6, 12, 14, 11}]];
```

```
untreatedSNPNsF = DeleteDuplicates[
   untreatedSNPNsF[[Flatten@Position[MemberQ[talliedNsFLUADMutList[[1 ;; 50, 1]], #] & /@
       untreatedSNPNsF[[All, 1]], True]]]];
```

The SNP data is then exported after being formatted for mutation signature analysis. The specific column header information can be found by executing “clin20[[1]]”:

```
Export["/path/to/Destination/mskFemaleNonSmokerToSigAna.csv",
 Prepend[Table[{i - 1, untreatedSNPNsF[[i, 1]], untreatedSNPNsF[[i, 2]],
    untreatedSNPNsF[[i, 3]], untreatedSNPNsF[[i, 4]], untreatedSNPNsF[[i, 5]],
    untreatedSNPNsF[[i, 6]], untreatedSNPNsF[[i, 7]]}, {i, 1, Length@untreatedSNPNsF}],
  {"", "Hugo_Symbol", "Tumor_Sample_Barcode", "Chromosome", "Start_Position",
   "Reference_Allele", "Tumor_Seq_Allele2", "Variant_Type"}]]
```

A similar process is used to gather the mutation signatures for the untreated US PTC and SKCM cohorts. Starting from a slightly modified clinical data file (unimportant columns deleted), we first determine the position of the untreated patients and then subsequently gather their patient IDs. Once all the untreated patient IDs are gathered, we then retain the mutation data from the maf files downloaded from Broad Firebrowse (see README.txt in the primary directory for details- like the Import function above, the FileNames function used below requires the path information pointing to the directory containing the MAF files). The SNP mutations are flagged and and those within the top 50 mutated cancer census gene panel are processed for mutation signature analysis.

TCGA Untreated SKCM Patients

Treatment naive patient IDs (indexed between row 2 and 226) are extracted using the treatment status file:

```
melNaiveIDList =
  Import["/path/to/Supplementary Software 1/Input Files/treatmentStatusSKCM.csv",
    "CSV"][[2 ;; 226, 1]];
```

Note: If a manifest file is present within the directory, use it to verify the contents in the directory; the manifest file should then be moved to another directory. The path information of the total melanoma patients are obtained:

```
totMelFiles = FileNames[
   "/path/to/gdac.broadinstitute.org_SKCM.Mutation_Packager_Raw_Calls.Level_
     3.2016012800.0.0/*.txt"];
```

Duplicate IDs exist. For the purpose of the analysis TCGA-ER-A2NF-06 and TCGA-ER-A19T-06 are used. The mutation percentages of the total SKCM cohort are calculated below:

```
In[ ]:= totMelFiles = Delete[totMelFiles, {{278}, {270}}];
```

```
In[ ]:= totalMelMutList = {};
Do[(
   tempImp = Rest@Import[totMelFiles[[i]], "TSV"];
   delVec = Join[Position[tempImp[[All, 9]], "Silent"], Position[tempImp[[All, 9]], "RNA"]];
   tempImp = Delete[tempImp, delVec];
   AppendTo[totalMelMutList, Union[tempImp[[All, 1]]]];
  ), {i, 1, Length@totMelFiles}]

In[ ]:= talliedMelMutList = ReverseSortBy[Tally[Flatten[totalMelMutList]], Last];

Export["/path/to/Destination/tcgaTotalSKCM_MutationCounts.csv", talliedMelMutList[[
   Flatten@Position[MemberQ[goisUS, #] & /@ talliedMelMutList[[All, 1]], True]]]]
```

Focus is shifted to calculate the mutation percentages for the naive treated SKCM patients.

```
naiveIndices =
   Flatten[Position[StringTake[StringSplit[totMelFiles, "/"][[All, -1]], 12], #] & /@
     melNaiveIDList];
```

The path information files containing the mutation data of the treatment naive SKCM patients is then obtained:

```
melanomaNaiveFiles = totMelFiles[[naiveIndices]];
```

Total mutation numbers of the treatment naive SKCM patients are obtained;

```
In[ ]:= naiveMelMutList = {};
Do[(
   tempImp = Rest@Import[melanomaNaiveFiles[[i]], "TSV"];
   delVec = Join[Position[tempImp[[All, 9]], "Silent"], Position[tempImp[[All, 9]], "RNA"]];
   tempImp = Delete[tempImp, delVec];
   AppendTo[naiveMelMutList, Union[tempImp[[All, 1]]]];
  ), {i, 1, Length@melanomaNaiveFiles}]

In[ ]:= talliedNaiveMelMutList = ReverseSortBy[Tally[Flatten[naiveMelMutList]], Last];
```

The mutations within the cancer gene census panel are then obtained:

```
canConTalliedNaiveMelMutList = talliedNaiveMelMutList[[
    Flatten@Position[MemberQ[goisUS, #] & /@ talliedNaiveMelMutList[[All, 1]], True]]];
```

Specific cancer gene census panel mutation information of the treatment naive SKCM patients is obtained:

```
In[ ]:= naiveMelToSigAna = {};
Do[(
   tempImp = Rest@Import[melanomaNaiveFiles[[i]], "TSV"];
   delVec = Join[Position[tempImp[[All, 9]], "Silent"], Position[tempImp[[All, 9]], "RNA"]];
   tempImp = Delete[tempImp, delVec];
   AppendTo[naiveMelToSigAna, tempImp[[Flatten[Position[
        MemberQ[canConTalliedNaiveMelMutList[[1 ;; 50, 1]], #] & /@ tempImp[[All, 1]], True]]]]]
  ), {i, 1, Length@melanomaNaiveFiles}]
```

The SNPs within the list above are identified and formatted for mutation signature analysis before being exported:

```
flatNaiveMelToSigAna = Flatten[naiveMelToSigAna, 1];
snpNaiveMelToSigAna =
   DeleteDuplicates[flatNaiveMelToSigAna[[Flatten@Position[SameQ["SNP", #] & /@
          flatNaiveMelToSigAna[[All, 10]], True], {1, 16, 5, 6, 11, 13, 10}]]];
toExport = {{"", "Hugo_Symbol", "Tumor_Sample_Barcode", "Chromosome",
     "Start_Position", "Reference_Allele", "Tumor_Seq_Allele2", "Variant_Type"}};
Do[(
   AppendTo[toExport, {i - 1, snpNaiveMelToSigAna[[i, 1]], snpNaiveMelToSigAna[[i, 2]],
     snpNaiveMelToSigAna[[i, 3]], snpNaiveMelToSigAna[[i, 4]], snpNaiveMelToSigAna[[i, 5]],
     snpNaiveMelToSigAna[[i, 6]], snpNaiveMelToSigAna[[i, 7]]}]
  ), {i, 1, Length@snpNaiveMelToSigAna}]

Export["/path/to/Destination/tcgaNaiveSKCM_ToSigAna.csv", toExport]
```

TCGA Untreated PTC Patients

The same steps highlighted above are performed to format the data for the TCGA papillary thyroid cancer (PTC) cohort for the purpose of conducting mutation signature analysis:

```
totThyFiles = FileNames[
    "/path/to/gdac.broadinstitute.org_THCA.Mutation_Packager_Raw_Calls.Level_
      3.2016012800.0.0/*"];

papThyIDList =
   Import["/path/to/Supplementary Software 1/Input Files/treatmentStatusPTC.csv",
     "CSV"][[All, 1]];

preFinalPapFileNames = totThyFiles[[
    Flatten[Position[StringTake[StringSplit[totThyFiles, "/"][[All, -1]], 12], #] & /@
      papThyIDList]]];

Position[preFinalPapFileNames,
  "/path/to/gdac.broadinstitute.org_THCA.Mutation_Packager_Raw_Calls.Level_
    3.2016012800.0.0/TCGA-EM-A2CS-01.maf.txt"]
```

Duplicate IDs exist. TCGA-EM-A2CS-06, TCGA-DE-A4MD-06, TCGA-J8-A4HW-06, TCGA-J8-A3YH-06, TCGA-J8-A3O2-06, TCGA-EM-A3SU-06, TCGA-E3-A3FQ-06, and TCGA-EM-A2P1-06 are used for subsequent

analysis. On the system that this code is being generated, TCGA-EM-A2CS-01, TCGA-DE-A4MD-01, TCGA-J8-A4HW-01, TCGA-J8-A3YH-01, TCGA-J8-A3O2-01, TCGA-EM-A3SU-01, TCGA-E3-A3FQ-01, and TCGA-EM-A2P1-01 are indexed within the brackets below. These IDs can be searched using the piece of code directly above this text using the Position function.

```
In[∘]:= finalPapFileNames =
  Delete[preFinalPapFileNames, {{192}, {309}, {341}, {305}, {237}, {115}, {232}, {268}}];

In[∘]:= totalPapMutList = {};
Do[(
  tempImp = Rest@Import[finalPapFileNames⟦i⟧, "TSV"];
  delVec = Join[Position[tempImp⟦All, 9⟧, "Silent"], Position[tempImp⟦All, 9⟧, "RNA"]];
  tempImp = Delete[tempImp, delVec];
  AppendTo[totalPapMutList, Union[tempImp⟦All, 1⟧]];
 ), {i, 1, Length@finalPapFileNames}]

papThyNaiveIDList = papThyIDList⟦2 ;; 106⟧;

In[∘]:= talliedPapMutList = ReverseSortBy[Tally[Flatten[totalPapMutList]], Last];

Export["/path/to/Destination/tcgaTotalPTC_MutationCounts.csv", talliedPapMutList⟦
  Flatten@Position[MemberQ[goisUS, #] & /@ talliedPapMutList⟦All, 1⟧, True]⟧]

naiveThyIndices =
  Flatten[Position[StringTake[StringSplit[totThyFiles, "/"]⟦All, -1⟧, 12], #] & /@
    Flatten[papThyNaiveIDList]];

papThyNaiveFiles = totThyFiles⟦naiveThyIndices⟧;

In[∘]:= naivePapThyMutList = {};
Do[(
  tempImp = Rest@Import[papThyNaiveFiles⟦i⟧, "TSV"];
  delVec = Join[Position[tempImp⟦All, 9⟧, "Silent"], Position[tempImp⟦All, 9⟧, "RNA"]];
  tempImp = Delete[tempImp, delVec];
  AppendTo[naivePapThyMutList, Union[tempImp⟦All, 1⟧]];
 ), {i, 1, Length@papThyNaiveFiles}]

In[∘]:= talliedNaivePapThyMutList = ReverseSortBy[Tally[Flatten[naivePapThyMutList]], Last];

canConTalliedNaivePapMutList = talliedPapMutList⟦
   Flatten@Position[MemberQ[goisUS, #] & /@ talliedPapMutList⟦All, 1⟧, True]⟧;
```

In[ ]:=
```
naivePapToSigAna = {};
Do[(
   tempImp = Rest@Import[papThyNaiveFiles⟦i⟧, "TSV"];
   delVec = Join[Position[tempImp⟦All, 9⟧, "Silent"], Position[tempImp⟦All, 9⟧, "RNA"]];
   tempImp = Delete[tempImp, delVec];
   AppendTo[naivePapToSigAna, tempImp⟦Flatten[Position[
        MemberQ[canConTalliedNaivePapMutList⟦1 ;; 50, 1⟧, #] & /@ tempImp⟦All, 1⟧, True]]⟧]
  ), {i, 1, Length@papThyNaiveFiles}]

flatNaivePapToSigAna = Flatten[naivePapToSigAna, 1];
snpNaivePapToSigAna =
   DeleteDuplicates[flatNaivePapToSigAna⟦Flatten@Position[SameQ["SNP", #] & /@
          flatNaivePapToSigAna⟦All, 10⟧, True], {1, 16, 5, 6, 11, 13, 10}⟧];
toExport = {{"", "Hugo_Symbol", "Tumor_Sample_Barcode", "Chromosome",
     "Start_Position", "Reference_Allele", "Tumor_Seq_Allele2", "Variant_Type"}};
Do[(
   AppendTo[toExport, {i - 1, snpNaivePapToSigAna⟦i, 1⟧, snpNaivePapToSigAna⟦i, 2⟧,
     snpNaivePapToSigAna⟦i, 3⟧, snpNaivePapToSigAna⟦i, 4⟧, snpNaivePapToSigAna⟦i, 5⟧,
     snpNaivePapToSigAna⟦i, 6⟧, snpNaivePapToSigAna⟦i, 7⟧}]
  ), {i, 1, Length@snpNaivePapToSigAna}]

Export["/path/to/Destination/tcgaNaivePTC_ToSigAna.csv", toExport]
```
